# Editorial: Behaviors, bias, and decision-making in health

**DOI:** 10.3389/fpsyg.2025.1667225

**Published:** 2025-09-08

**Authors:** Silvia Riva, Marcelle Fernandes, Loredana Minini, Paola Iannello

**Affiliations:** ^1^Department of Psychology, School of Allied Health and Life Sciences, St Mary's University, Twickenham, London, United Kingdom; ^2^Department of Psychology, Universita Cattolica del Sacro Cuore, Milan, Italy

**Keywords:** health, behavior, bias, decisions, behavioral insights

The nexus of mental health, cognitive functions, and health-related behavior presents a rich field for applied psychological research, shedding light on how psychological, emotional, and social factors influence health decisions, be they social, clinical, or related to individual strategies. The research call, *Behaviors, bias, and decision-making in health*, was intentionally created to make a meaningful contribution in this applied domain. Thirteen interdisciplinary contributions explore the cognitive and emotional factors, mechanisms, and potential interventions that shape behavior in health contexts.

One of the key contexts in which to explore health-related behaviors has been the extraordinary case of the COVID-19 pandemic. A Korean study revealed the potential adaptive effects of the *maximization* personality trait, the tendency to carefully evaluate options in pursuit of the best possible outcome, during the pandemic (Jun et al.). Certain aspects of maximization may promote wellbeing under stress, particularly when mediated by effective coping mechanisms such as cognitive reappraisal and preventive behaviors (e.g., wearing masks).

Importantly, one key lesson from the pandemic is that such knowledge should not be forgotten but rather integrated into everyday health and preventive policy. Sustained public health messaging and targeted interventions to support long-term protective behaviors are crucial, especially in anticipation of possible future outbreaks. This is one of the main practical implications of Luo et al.'s study.

However, although people in post-pandemic China retain a good understanding of protective measures, actual adherence to these behaviors has declined compared to the peak of the pandemic. This decline appears to be linked to a reduced sense of vulnerability and a prevailing belief that the pandemic is now behind us, elements that are particularly important to address in preventive contexts (Luo et al.). At the same time, such an exceptional event has the potential to shift some behaviors in a more health-conscious direction. For instance, another study suggested that the pandemic acted both as a barrier and a catalyst: urban residents encountered restricted access to safe walking and cycling infrastructure and pandemic-related anxieties, but at the same time, the crisis triggered a shift toward sustainable transport, as public awareness of health and environmental benefits increased (Du S. et al.). These ambivalent effects illustrate the dynamic interplay between environmental constraints and motivational shifts.

Building on this, this psychological framing is equally relevant in the context of chronic disease management, where sustained behavioral change is crucial. Chronic disease management demands more than clinical instruction; it relies on individuals' cognitive and emotional capacity to transform knowledge into sustained action. Across several studies in this Research Topic, psychological mechanisms such as health literacy, self-efficacy, illness perception, and stigma consistently emerge as critical drivers of health-related behavior. In China, research on hypertension and rheumatoid arthritis shows that it is not merely what patients know, but how they interpret and internalize their illness that shapes their ability to engage in effective self-management (Liu T. et al.; Liu Y. et al.). These cognitive and emotional variables mediate the transition from understanding to action, influencing medication adherence, symptom monitoring, and lifestyle change.

This pattern is echoed in studies on diabetes and chronic heart failure. Patients with type 2 diabetes often underestimate the long-term consequences of their condition, delaying care-seeking and underutilizing available health resources due to low perceived threat and high perceived barriers (Du Q.-h. et al.). Similarly, individuals with chronic heart failure frequently avoid recommended physical activity due to Kinesiophobia, a fear of movement that stems from emotional distress and perceived vulnerability (Xiang et al.). In both cases, behavioral disengagement is not the result of ignorance, but of underlying belief systems and emotional responses.

In addition, health-related decisions and behaviors are closely linked to stress and its impact on body functioning. Zhang et al. show that poor sleep is not just a physiological response to stress but also influenced by maladaptive coping strategies like rumination and excessive smartphone use. Similarly, Giaume et al. found that first responders in high-stress simulations experienced anticipatory anxiety and reduced body awareness, affecting their performance and recovery. Both studies highlight the need to address emotional regulation and behavioral habits to improve health outcomes in high-stress environments.

Finally, other contributions have offered valuable suggestions for the promotion of interventions. Bientzle et al. show that interventions rooted in storytelling—like narrative writing and narrative reading—can promote empathic concern and reduce stigma toward individuals who engage in socially disapproved health behaviors (e.g., smoking while pregnant). This study found that the less time-consuming technique of narrative reading is as effective as narrative writing in increasing empathic concern, perspective-taking, and attitudinal change. This opens the door to scalable, time-efficient formats for empathy-based health communication. The importance of social support is also emphasized in the study by Pan et al.. These authors report that perceived social support had a positive effect on the vision-related quality of life of elderly individuals with dry eye disease. Patients with social support had greater health outcomes; specifically, social support improved the patients' illness perception and confrontational copying style, suggesting a potential wider role of social support for healthcare interventions. These results reinforce the role of emotional and relational factors in sustaining healthy behaviors, especially in older populations. They also suggest the benefit of integrating social resources into personalized care pathways.

Finally, two conceptual contributions address broader behavioral frameworks. A systematic review of nudging strategies in chronic obstructive pulmonary disease management highlights how behavioral science can improve adherence and outcomes (Wu et al.). This study explored the role of nudges such as social influence, gamification, reminders, and feedback on a range of health behaviors. Medication adherence was improved by both reminders and feedback on mobile devices. Additionally, reminders through text materials also improved inhalation techniques and vaccination in patients. A discussion paper by Bonazza et al. examines patient-centered care at the end-of-treatment. Ethical and psychological tensions that impact shared decision-making were examined, in particular in instances when patients' autonomy challenges best clinical treatment and when proposed treatment challenges the patient's preferences. The study contributes to the ongoing discourse on the balance between paternalism and autonomy in medical decision making.

Together, these studies highlight a crucial insight: effective public health and clinical interventions must move beyond information provision. Addressing cognitive distortions, emotional readiness, and belief systems is essential to supporting long-term behavioral change. A psychologically informed approach can better align interventions with the realities of how individuals experience and respond to illness, thus helping bridge the gap between knowledge and action in diverse health contexts.

